# Quality assessment of services provided by health centers in Mashhad, Iran: SERVQUAL versus HEALTHQUAL scales

**DOI:** 10.1186/s12913-021-06405-4

**Published:** 2021-04-28

**Authors:** Tahereh Sharifi, Seyede-Elahe Hosseini, Saeed Mohammadpour, Javad Javan-Noughabi, Hosein Ebrahimipour, Elahe Hooshmand

**Affiliations:** 1 Health Sciences Research Center, Torbat Heydariyeh University of Medical Sciences, Torbat Heydariyeh, Iran; 2 School of Health Management and Information Sciences, Isfahan University of Medical Sciences, Isfahan, Iran; 3 School of Management and Medical Information, Iran University of Medical Sciences, Tehran, Iran; 4 Social Determinants of Health Research Center, Mashhad University of Medical Sciences, Mashhad, Iran

**Keywords:** Service quality, Health care evaluation, Health services research, Primary health care, Health-care providers

## Abstract

**Background:**

Primary health care is the entry point to the health-care system and regarded as an essential step to achieving universal health coverage. The present study aimed at evaluating the quality of health-care services provided in health centers in Mashhad, Iran.

**Methods:**

This was a cross-sectional study implemented among 200 health service users who were referring to four health centers in Mashhad during January to June 2019. The quality of services in health centers was evaluated with the SERVQUAL and HEALTHQUAL models. Data was analyzed by employing paired *t*-test and independent sample *t*-test using SPSS version 16 software. The Levene test was used for examining the equality of variance (homogeneity). Significance level of all the tests was considered when *p* ≤ 0.05.

**Results:**

According to the results of SERVQUAL questionnaire, the average scores of health service users’ expectations and perceptions were 4.97 and 3.26, respectively, and the quality gap in the provided services was equal to − 1.7. Based on HEALTHQUAL questionnaire, the average scores of health service users’ perception and expectations were 4.72 and 3.25, respectively, and the quality gap in the provided services was equal to − 1.16. Empathy was the highest quality dimension (− 2.019) based on SERVQUAL model, and efficiency dimension was the highest based on HEALTHQUAL model (− 1.761).

**Conclusions:**

The findings of the current study showed a negative gap between the service users’ expectations and perceptions in both models. Therefore, the results of this study helps the health managers and policymakers to plan effective interventions for improving the provided services emphasizing the dimensions with the wider gaps.

**Supplementary Information:**

The online version contains supplementary material available at 10.1186/s12913-021-06405-4.

## Background

In Alma-Ata declaration (1978), primary health care (PHC) was introduced as the key element to achieve “Health for all by the year 2000” [1]. PHC is an important strategy that provides universal health care coverage in order to increasing health equity and health promotion [2]. In Iran, health-care services are provided at three levels: primary, secondary, and tertiary. Health centers are responsible for providing primary health care services such as promotive, preventive and curative, and refer only those cases that need more specialized services including secondary and tertiary care [3].

Health centers are the first point of contact between the society and health system [4]. Thus, providing appropriate and quality health-care services in these centers will prevent unnecessary referrals and consequently additional costs. Also, high quality PHC services can lead to increased satisfaction, trust and confidence, speed of service delivery and cost-effectiveness of health-care services and decrease waiting time as well [5–8]. The world health organization (WHO) has emphasized the importance of quality in providing health-care services and declared the access to quality health care for all as the way of achieving sustainable development [6]. Also in Iran, provision of quality health services has been focused in the country’s upstream documents such as the general health policies in the national five-year development plan [9, 10].

A way of evaluating service quality is the use of SERVQUAL model that has been proposed by Parasuraman et al. based on the gap theory of service quality [11]. This questionnaire has been employed in previous studies for quality assessment of services [12–16]. In this tool, health-care service quality is evaluated by comparing the customers’ expectations and perceptions from different aspects (physical and tangible dimensions of service, assurance, responsiveness, service reliability, and empathy) [11, 17]. Although this tool has been utilized in the area of health, using a special questionnaire for health-care providers beside the general questionnaire such as SERVQUAL can introduce more realistic results of the comparison. Therefore, by conducting studies in health-care context in Iran, a tool called HEALTHQUAL was designed to give a better image about the quality of health-care services [18, 19]. This questionnaire has been previously employed in several studies to evaluate the quality of health-care services [20, 21].

Various studies have investigated service quality and addressed the quality gap in services provided in comprehensive health service centers and hospitals. The results of these studies suggest that there is an improper responsiveness of health system to people’s health priorities and raised health-care costs [12–16, 22, 23]. Despite many studies carried out on quality of health-care services in Iran, there is a lack of evidence for health-care quality in Mashhad. Mashhad is a populous city inhabited with more than 3 million people located in the northeast of Iran. Every year, almost 2 million pilgrims from all around the world travel to Mashhad to practice religious rituals. Accordingly, health centers play a central role in Mashhad and they face many challenges in their strategies to provide PHC. On the other hand, there are few quality assessment studies that have been undertaken using HEALTHQUAL model.

The present study aimed at evaluating the quality of health services provided in health centers using two questionnaires, SERVQUAL and HEALTHQUAL. The findings of this study granted evidences regarding the quality gaps in primary health care. These evidences help researchers in health field, and also policymakers to develop effective interventions for eliminating the recognized gaps.

## Methods

This was a cross-sectional study implemented among health service users who were referring to four health centers in Mashhad, the second largest city in Iran and the capital of Razavi Khorasan province, during January to June 2019.

The sample size was 200, which has been determined using the following formula:

$$ {\displaystyle \begin{array}{c}n={\mathrm{Z}}_{1\hbox{-} \upalpha /2}^2\kern0.5em \ast \kern0.5em {\upsigma}^2\kern0.5em /\kern0.5em {\mathrm{d}}^2\\ {}\mathrm{Sample}\kern0.5em \mathrm{size}\kern0.5em \mathrm{formula}\end{array}} $$

Where, n is the sample size, z is 1.96 for 95% confidence level; σ is the standard deviation which is equal to 0.35, and d is the margin of error which is equal to 0.05.

There are five main comprehensive health centers in Mashhad city (No. 1, 2, 3, and 5 and Samen Health Center). We selected the four centers that cover the largest population. Then, 50 people from each center were randomly selected based on the inclusion criteria. It should be noted that the sample size was evenly divided between the centers due to the relatively similar volume of population covered by the four selected centers. Health service users aged 18 years old and more who were willing to participate in this study and able to speak Persian were included. People who refused to participate in the study were excluded. Participants completed questionnaires immediately after receiving the service.

In this study, we used both of SERVQUAL and HEALTHQUAL models in order to evaluate the quality of services in health centers, and to compare the results of two methods. The SERVQUAL and HEALTHQUAL methods identify the gaps between customers’ expectations and perceptions of service quality.

SERVQUAL model was developed by Parasuraman, Zeithaml & Berry [11], which consists of five dimensions and 22 questions:

1. Tangibles (with 4 questions about physical, equipment, and appearance of facilities)
2. Reliability (includes 5 questions about ability to provide a promised service dependably and accurately)
3. Responsiveness (encompasses 4 questions about willing to help service users promptly and efficiently)
4. Assurance (embraces 4 questions about manner and expertise of employee and ability the establishing trust and confidence in customers)
5. Empathy (with 5 questions about individualized attention of organization to its clients).

The reliability of the instrument was measured by Cronbach alpha which was 0.96 for perceptions and 0.93 for expectations [24].

We also used HEALTHQUAL model that has been applied by Mosadeghrad to evaluate health service quality [19]. This questionnaire includes 4 domains and 30 questions:

1. Environment (embraces 11 questions about physical facilities, buildings, equipment)
2. Empathy (with 12 questions about interactions between healthcare providers and service users)
3. Efficiency (composed of 3 questions about factors such as waiting time, speed of service delivery and cost-effectiveness of healthcare services)
4. Effectiveness (encompasses 4 questions about expected goals of service users).

The reliability was checked using Cronbach alpha coefficient which was 0.96 the HEALTHQUAL questionnaire [19].

Both questionnaires were completed by the participants two times, once before receiving the service to evaluate their expectations (evaluation of desirable situation), and another time after receiving the service to evaluate their perceptions (evaluation of current situation). The gap between expectations and perceptions defined as services quality, as the negative gap means low quality and the positive one indicates high quality.

Both questionnaires were on a five-point Likert scale that ranges from: 1 = strongly disagree, 2 = disagree, 3 = uncertain, 4 = agree, to 5 = strongly agree.

Data were analyzed using SPSS version 16 by applying descriptive (frequency, percentage, mean and standard deviation) and analytical (paired *t*-test and independent *t*-test) statistics. Paired *t*-test was used to compare the mean scores of expectations and perceptions in each model. Independent *t*-test was used to compare the mean scores of quality gap according to the gender (male or female), age (≤40 or > 40 years old), marital status (single or married) and education level (diploma & lower or Bachelor). Also, the Levene test was employed for examination of the equality of variance homogeneity.

### Ethical consideration

This approval of our study was granted by the Ethics Committee of Torbat Heydarieh University of Medical Sciences [The code of Ethics: IR.THUMS.REC.1396.43]. All participants have signed the written informed consent form for the various aspects of data collection. Confidentiality and privacy were assured for all study participants. All methods were carried out in accordance with relevant guidelines and regulations under Ethics approval and consent to participate.

## Results

Table 1 shows that among the 200 participants surveyed, the vast majority of participants were below 40 years old (*n* = 178, 90%), women (*n* = 181, 91%), married (*n* = 196, 98%) and graduates university education (*n* = 110, 56%).

**Table 1:** Sociodemographic characteristic of service users (*N* = 200)

**Variation**	**Number**	**Percentage**
**Age**		
≤ 40	179	90
> 40	21	10
**Sex**		
Men	19	9
Women	181	91
**Marital status**		
Married	196	98
Single	4	2
**Education level**		
University education	110	56
Non-university education	90	44

As exhibited in Table 2, there was a significant negative quality gap in all 5 SERVQUAL dimensions (*p* < 0.001). The highest and the lowest gaps in services quality were related to the dimensions of “Empathy” (− 2.019) and “Tangibles” (− 1.483). Moreover, total services quality gap was (− 1.707), indicating that the provided services did not meet the expectations of service users.

**Table 2:** Mean and standard deviation of the service user perceptions, expectations, and service gaps in SERVQUAL model

**Dimensions and items**	**Expectation**	**Perception**	**Quality Gap**	**t-test**	***P*-Value**
	(Mean ± SD)	(Mean ± SD)	(Mean ± SD)		
**Tangibles**	4.981 ± 0.086	3.497 ± 0.535	− 1.483 ± 0.545	−38.452	0.000*
**Reliability**	4.976 ± 0.105	3.237 ± 0.505	− 1.738 ± 0.535	−45.930	0.000*
**Responsiveness**	4.98 ± 0.104	3.487 ± 509	−1.492 ± 0.525	−40.199	0.000*
**Assurance**	4.964 ± 0.128	3.163 ± 0.856	− 1.801 ± 0.867	− 29.372	0.000*
**Empathy**	4.972 ± 0.109	2.953 ± 1.235	−2.019 ± 1.239	− 23.042	0.000*
**Total Service Quality**	4.974 ± 0.086	3.267 ± 0.546	−1.707 ± 0.558	− 43.233	0.000*

*Statistically significant at 0.05 level

Table 3 indicates that the expectation score was higher than the perception score in all HEALTHQUAL dimensions. The highest gaps in services quality were associated with dimensions of “Empathy” (− 1.6 ± 0.106), followed by “Environment” and “Effectiveness” The lowest gaps in services quality demonstrated in “Efficiency” dimension (− 0.698 ± 0.638). In HEALTHQUAL model, total services quality gap was (− 1.104).

**Table 3:** Mean scores and standard deviation of the service user perceptions, expectations, and service gaps in HEALTHQUAL model

**Dimensions and items**	**Expectation**	**Perception**	**Quality Gap**	**t-test**	***P*-Value**
	(Mean ± SD)	(Mean ± SD)	(Mean ± SD)		
**Environment**	4.832 ± 0.066	3.736 ± 0.535	− 1.096 ± 0.980	−42.436	0.001*
**Empathy**	4.769 ± 0.112	3.169 ± 0.505	− 1.6 ± 0.106	− 41.106	0.000*
**Efficiency**	4.652 ± 0.225	3.954 ± 901	− 0.698 ± 0.638	−38.226	0.001*
**Effectiveness**	4.762 ± 0.137	3.742 ± 0.823	− 1.02 ± 0.724	− 43.309	0.000*
**Total Service Quality**	4.723 ± 0.020	3.256 ± 0.789	− 1.104 ± 0.567	−30.697	0.000*

*Statistically significant at 0.05 level

A statistical evaluation using student *t*-test in Table 4 showed that there was no significant difference in SERVQUAL and HEALTHQUAL scores according to age, sex and educational status (*p* > 0.05). However, we found that there was a statistically significant difference in SERVQUAL scores between the two groups of marital status (*p* < 0.05).

**Table 4:** Associations between service users’ demographic characteristics and the mean of quality gaps: HEALTHQUAL and SERVQUAL scores

**Characteristics**	**Quality gap based on HEALTHQUAL model**					**Quality gap based on SERVQUAL model**				
	**LEVENE’S Test for Equality of Variances**	***F***	***P*-value**	**Independent Samples Test**	***P*-value**	**LEVENE’S Test for Equality of Variances**	***F***	***P*-value**	**Independent Samples Test**	***P*-value**
**Gender**	Equal variances assumed	0.01	0.752	− 0.414	0.679	Equal variances assumed	0.08	0.778	−0.732	0.465
	Equal variances not assumed			−0.426	0.674	Equal variances not assumed			−0.669	0.511
**Age**	Equal variances assumed	0.419	0.518	−1.058	0.291	Equal variances assumed	0.105	0.746	1.352	0.178
	Equal variances not assumed			−1.059	0.3	Equal variances not assumed			1.454	0.158
**Marital status**	Equal variances assumed	0.033	0.857	−0.086	0.931	Equal variances assumed	0.327	0.568	2.016	0.045*
	Equal variances not assumed			−0.101	0.926	Equal variances not assumed			1.53	0.222
**Education level**	Equal variances assumed	0.622	0.431	0.799	0.425	Equal variances assumed	0.095	0.759	−1.277	0.203
	Equal variances not assumed			0.813	0.417	Equal variances not assumed			−1.268	0.206

*Statistically significant at 0.05 level

## Discussion

The main aim of the present study was to evaluate the quality of provided services in health centers in Mashhad using SERVQUAL and HEALTHQUAL models.

The findings of the current study showed a negative gap between the service users’ expectations and perceptions in both model. It means that quality of the delivered PHC services was lower than what the service users expected. The quality gap in the provided services was (− 1.7) based on SERVQUAL model and (− 1.1) according to HEALTHQUAL model. This result was consistent with most of the previous studies on health-care quality assessment. The quality gap in the provided health-care services in urban primary health care centers in Kermanshah province of Iran was reported about (− 1.4) based on SERVQUAL model [25]. Evaluation of health-care service quality via SERVQUAL scale in a hospital in Turkey displayed that the quality gap in the provided health-care services was (− 2.24) [13]. Quality of health-care services in public and private hospitals was compared in a study performed in Peshawar, Pakistan. In that study, the quality gap based on SERVQUAL model was (− 1.85) for public hospitals and (− 0.86) for private hospitals [26]. Mosadeghrad and Sokhanvar revealed that the quality gaps in the provided health-care services were (− 0.57) and (− 0.58), respectively in two studies conducted based on HEALTHQUAL model [21]. Nemati et al., in a study aimed to compare the Iranian university and non-university hospitals service quality based on the HEALTHQUAL model, found that the quality gap was − 0.42 and − 0.64 for university and non-university hospitals respectively [20]. The quality gap reported in these studies was less than the value mentioned in the present study, and this can be attributed to the difference between the population and study setting (hospital vs. health centers). Also, the type of provided services in hospitals is different from that in health centers.

SERVQUAL results denoted that service users’ expectations have been at a high level in all dimensions (environment, human interaction, efficiency, and effectiveness), and the highest score has been reported in “Tangibility” and “Responsiveness” dimensions (4.98). A study conducted by Pekkaya et al. showed a similar result. In this study, the highest expectation score was related to “Tangibility” (8.15) [13]. In HEALTHQUAL model, the highest average score of expectations was related to “Environment” (4.832). This result was in consistent with Nemati et al. study. This study showed that patients at hospitals paid more attention to “environment” dimension based on HEALTHQUAL model [20]. Since Tangibility dimension in SERVQUAL refers to physical and observable services same as the Environment dimension in HEALTHQUAL, it can be approved that the two questionnaires present had almost similar results in terms of service users’ expectations. Therefore, service users’ expectations mainly focused on the physical space, staff appearance, and modern equipment. Health care is one of the complex services and due to information asymmetry between service users and health-care providers, the service users cannot evaluate the quality service itself, accordingly, they assess that based on tangible items such as the physical environment and equipment.

The results of SERVQUAL model in terms of perceptions indicated that the highest score was related to “Tangible” dimension (3.49). The results reported in several studies exhibited that the “Assurance” was the highest-performing dimension [25, 27]. Another study discovered that the highest perception score was associated with “Reliability” dimension [28, 29]. Interestingly, none of these studies was consistent with the present study, as such, the possible explanation of the difference in results can be due to the heterogeneous study setting and the diverse array of health-care services provided. For example, the study conducted by Karydis has specifically focused on dental health care [27], however, a quality assessment study carried out by Peprah et al. showed similar results [30]. The results of HEALTHQUAL model showed that the highest average score of perceptions was related to “Efficiency” dimension (3.954). This finding of our study was inconsistent with the other studies. Two studies evaluated the quality of services in hospitals using HEALTHQUAL instrument. In these studies, the lowest average score of perceptions was related to “Efficiency” dimension [20, 21]. In HEALTHQUAL model, efficiency refers to factors such as waiting time, speed of service delivery and cost-effectiveness of healthcare services. Difference between the population and study setting is one of the main reasons for different results.

The highest quality gaps were observed in “Empathy” dimension according to SERVQUAL and HEALTHQUAL model. Empathy stands for understanding the needs of service users, and has a great role in communication with them. In HEALTHQUAL model, empathy includes questions about interactions between healthcare providers and service users. Different studies reported similar results [28, 31] Nemati et al. in their study showed that the highest quality gap was related to “Empathy” and “Environment” dimensions [20]. In study performed by Mosadeghrad and Sokhanvar [21], “Efficiency” was rated as the least quality gap, and this was in line with the present study. The low expectation in “Efficiency” dimension according to service users’ view can be due to the fact that services provided in health-care centers are for-free, and also with low costs in the public hospitals because of the health insurance. These merits contribute in financial protection of service users, and this might lead to less expectation in this dimension.

One of the research limitations was lack of investigation of staff viewpoints. Hence, it is suggested to survey their viewpoints in future studies and to employ qualitative methods for identifying the causes beyond quality gaps, and attempting to come up with appropriate solutions to decrease or even eliminate the gap in health-care centers where the provided services are of low quality.

## Conclusion

The results of the present research showed that the service quality is at a lower level than people’s expectations. In this way, improving the quality of services in primary health care can result in more effective prevention services and ultimately raise the level of community health status. The highest quality gaps were noticed in “Empathy” dimension according to SERVQUAL and HEALTHQUAL model, and this draws the attention of policymakers to set a reform plan which might include improvement of “Empathy” dimension, paying attention to patient when utilizing health services, proper training of staff on how to deal with clients professionally to reduce the quality gap.

## Supplementary Information

**Additional file 1.**

## Data Availability

The datasets used and/or analysed during the current study are available from the corresponding author on reasonable request.

## Abbreviations

PHC: Primary Health Care
WHO: World Health Organization
