# Supplementary material for: Quality assessment of services provided by health centers in Mashhad, Iran: SERVQUAL versus HEALTHQUAL scales
Source: BMC Health Serv Res. 2021 Apr 28;21:397. doi: 10.1186/s12913-021-06405-4 (PMC8082605; doi:10.1186/s12913-021-06405-4)
Supplement: Supplementary file 1 — Additional file 1. [file 12913_2021_6405_MOESM1_ESM.docx]

**Quality assessment of services provided by primary health care centers**

**Age:** **Sex:** Male Female

**Marital status:** Married Single **Education:** university education Non-university education

**Model 1: SERVQUAL**

| Dimension | Item | Expectation | | | | | Perception | | | | |
| --- | --- | --- | --- | --- | --- | --- | --- | --- | --- | --- | --- |
|  |  | 1 | 2 | 3 | 4 | 5 | 1 | 2 | 3 | 4 | 5 |
| Tangible | Up-to-date equipment and facilities |  |  |  |  |  |  |  |  |  |  |
|  | Appealing physical environment and good signs, symbols and artifacts |  |  |  |  |  |  |  |  |  |  |
|  | Well-dressed and neat employees and physicians |  |  |  |  |  |  |  |  |  |  |
|  | Appealing consumable elements used in cure and serve to patients |  |  |  |  |  |  |  |  |  |  |
| Reliability | Doing the promised service on time |  |  |  |  |  |  |  |  |  |  |
|  | Interesting to solve your problems |  |  |  |  |  |  |  |  |  |  |
|  | Doing everything right at the first time |  |  |  |  |  |  |  |  |  |  |
|  | Fulfilling the promised service at promised time |  |  |  |  |  |  |  |  |  |  |
|  | Keeping accurate records and documents |  |  |  |  |  |  |  |  |  |  |
| Responsiveness | Announce the exact time for providing services |  |  |  |  |  |  |  |  |  |  |
|  | Receiving fast and promptly service |  |  |  |  |  |  |  |  |  |  |
|  | Health center staff are Willing to help |  |  |  |  |  |  |  |  |  |  |
|  | Availability of staff while needed and demanded |  |  |  |  |  |  |  |  |  |  |
| Assurance | Trustful behavior of staff |  |  |  |  |  |  |  |  |  |  |
|  | Sense of security and comfort in transaction with staff |  |  |  |  |  |  |  |  |  |  |
|  | Polite and humble staff |  |  |  |  |  |  |  |  |  |  |
|  | staff are knowledgeable enough to answer your questions |  |  |  |  |  |  |  |  |  |  |
| Empathy | Individual attention to patients |  |  |  |  |  |  |  |  |  |  |
|  | Have convenient hours in health care center |  |  |  |  |  |  |  |  |  |  |
|  | Understanding the patients’ specific needs |  |  |  |  |  |  |  |  |  |  |
|  | provide services in according to the interests of you |  |  |  |  |  |  |  |  |  |  |
|  | Pay attention to all patients equally irrespective of their social status |  |  |  |  |  |  |  |  |  |  |

**Model 2: HEALTHQUAL**

| No | Item | Expectation | | | | | Perception | | | | |
| --- | --- | --- | --- | --- | --- | --- | --- | --- | --- | --- | --- |
|  |  | 1 | 2 | 3 | 4 | 5 | 1 | 2 | 3 | 4 | 5 |
| 1 | Easy and fast access to the health center and needed service |  |  |  |  |  |  |  |  |  |  |
| 2 | Clean health center environment (corridors, waiting room, vaccination room, toilets, etc) |  |  |  |  |  |  |  |  |  |  |
| 3 | Feeling comfortable in the health center (sufficient space, no noise, etc) |  |  |  |  |  |  |  |  |  |  |
| 4 | Affordable access to health services (Financial access) |  |  |  |  |  |  |  |  |  |  |
| 5 | Easy and fast access to acceptable services (conceptual access) |  |  |  |  |  |  |  |  |  |  |
| 6 | Easy and timely access to needed services |  |  |  |  |  |  |  |  |  |  |
| 7 | Easy access to entertainment facilities (TV, newspaper, magazine, refrigerator, etc) |  |  |  |  |  |  |  |  |  |  |
| 8 | Modern and advanced equipment |  |  |  |  |  |  |  |  |  |  |
| 9 | Easy and fast access to clean, orderly, and disciplined staff when needed |  |  |  |  |  |  |  |  |  |  |
| 10 | High experience and expertise of health center staff |  |  |  |  |  |  |  |  |  |  |
| 11 | Clarity and comprehensibility of health center instructions and brochures |  |  |  |  |  |  |  |  |  |  |
| 12 | Polite, courteous and friendly behavior of health care staff and their work commitment |  |  |  |  |  |  |  |  |  |  |
| 13 | Health center staff being trustworthy and making people feel comfortable to communicate with them |  |  |  |  |  |  |  |  |  |  |
| 14 | Compassionate and patient behavior of health center staff and spending time to help the patients and solve their problems |  |  |  |  |  |  |  |  |  |  |
| 15 | Having supportive and caring staff responsive to patients’ individual needs and expectations and being flexible in meeting them |  |  |  |  |  |  |  |  |  |  |
| 16 | Staff paying attention to patients as human beings and respecting their opinions, requests, and expectations |  |  |  |  |  |  |  |  |  |  |
| 17 | Staff being respectful of patients’ independence and private space and their medical secrets |  |  |  |  |  |  |  |  |  |  |
| 18 | The ability of staff to convey accurate information to admitted patients in a comprehensible way |  |  |  |  |  |  |  |  |  |  |
| 19 | The ability of staff to explain technical information for patients in an understandable way |  |  |  |  |  |  |  |  |  |  |
| 20 | Staff making it possible for patients to participate in the treatment program; Staff considering the opinions of patients in the program |  |  |  |  |  |  |  |  |  |  |
| 21 | Staff behaving respectfully toward patients’ guardians and visitors; Staff making it possible for patient guardians to participate in the treatment program |  |  |  |  |  |  |  |  |  |  |
| 22 | Staff providing the necessary guidance and instructions to patients about treatment Process and referral to other level if needed |  |  |  |  |  |  |  |  |  |  |
| 23 | Cooperation and teamwork among the staff of different wards |  |  |  |  |  |  |  |  |  |  |
| 24 | The value of the services received for the cost paid |  |  |  |  |  |  |  |  |  |  |
| 25 | Speed of health center services |  |  |  |  |  |  |  |  |  |  |
| 26 | Short waiting time and minimum delay in services (providing services according to the planned schedule) |  |  |  |  |  |  |  |  |  |  |
| 27 | Achieving the expected results with acceptable outcomes in terms of improvement in physical and mental health |  |  |  |  |  |  |  |  |  |  |
| 28 | Relief from pain |  |  |  |  |  |  |  |  |  |  |
| 29 | Receiving no harm in the health center (safety in providing services) |  |  |  |  |  |  |  |  |  |  |
| 30 | Receiving complete and comprehensive services (referral of the patient to other health care levels and specialties if needed) |  |  |  |  |  |  |  |  |  |  |
